# Single/Multi-Network Conductive Hydrogels—A Review

**DOI:** 10.3390/polym16142030

**Published:** 2024-07-16

**Authors:** Nahid Hasan, Md Murshed Bhuyan, Jae-Ho Jeong

**Affiliations:** Department of Mechanical, Smart and Industrial Engineering (Mechanical Engineering Major), Gachon University 1342, Seongnam-si 13120, Republic of Korea; nahid237@gachon.ac.kr

**Keywords:** conductive hydrogel, self-healing, gamma radiation, stimuli-responsive, double/triple network

## Abstract

Hydrogels made from conductive organic materials have gained significant interest in recent years due to their wide range of uses, such as electrical conductors, freezing resistors, biosensors, actuators, biomedical engineering materials, drug carrier, artificial organs, flexible electronics, battery solar cells, soft robotics, and self-healers. Nevertheless, the insufficient level of effectiveness in electroconductive hydrogels serves as a driving force for researchers to intensify their endeavors in this domain. This article provides a concise overview of the recent advancements in creating self-healing single- or multi-network (double or triple) conductive hydrogels (CHs) using a range of natural and synthetic polymers and monomers. We deliberated on the efficacy, benefits, and drawbacks of several conductive hydrogels. This paper emphasizes the use of natural polymers and innovative 3D printing CHs-based technology to create self-healing conductive gels for flexible electronics. In conclusion, advantages and disadvantages have been noted, and some potential opportunities for self-healing single- or multi-network hydrogels have been proposed.

## 1. Introduction

Ordinary conductors are not affordable for flexible devices and medical sciences such as tissue engineering and drug delivery. Different polymers are extensively used in various fields as moisture detection sensors [1], water pollutant adsorbents [2], and conductors [3]. CHs are three-dimensional hydrophilic networks with properties such as electrical conductivity, high toughness, self-recoverability, freezing resistance, stimuli-responsiveness, stretchability, self-healing, transparency, and strain sensitivity [4]. Many of those properties are absent in metal conductors, which privilege hydrogel conductors to be incorporated into electrical devices [5]. There are still some challenges and limitations for conductive hydrogels, such as low mechanical strength, poor stability, and complex fabrication methods. However, researchers have been developing new strategies to improve the performance and functionality of conductive hydrogels, such as designing novel molecular structures, introducing additional components, creating double networks, and applying external stimuli. These approaches can endow conductive hydrogels with multiple functionalities, such as self-healing, super toughness, anti-freezing, antibacterial, and stimulus responsiveness. Moreover, these multifunctional CHs can be integrated with other devices, such as strain sensors, supercapacitors, touch panels, triboelectric nanogenerators, and bioelectronic devices, to achieve smart wearable devices with high performance and versatility. In this review, we summarize the recent advances in preparing and applying multifunctional conductive hydrogels for smart wearable devices and provide some perspectives on the future development of this promising field [6]. A few conducting organic compounds including 7,7,8,8-tetracyanoquinodimethane (TCNQ) [7], tetrathiafulvalene (TTE) [8], bis(ethylenedithiolo) tetrathiafulvalene (BEDT-TTF), and conducting polymers such as polyaniline (PANI) [9], polypyrrole (PPy) [10], poly(3,4-ethylenedioxythiophene: polystyrene sulfonate) (PEDOT:PSS) [11], polythiophene (PTh) [12], phenylene vinylene (PPV) [13], and polycarbazole (PC) [14] are available to use in various fields. The structure and properties of the above-mentioned compounds are listed in Table 1. Those conductive materials can be fabricated together to produce CHs, which are used in soft electronics [15], energy storage devices [16], touch panels [17], biomedicine, actuators [14], wearable strain sensors [18], textile engineering [19], drug delivery systems [20], and tissue engineering [12]. However, most of the hydrogels show a lack of self-healing, sufficient mechanical strength, recoverability, and conductivity [21]. To overcome the drawbacks of the presently available conductive hydrogels, suitable double-network hydrogels can be brought into the research. Double-network hydrogels are soft and tough materials with two contrasting polymeric networks with extra mechanical strength and toughness [22]. Recently, many double-network hydrogels have been synthesized using natural polymers, but they still lack better conductivity and strong mechanical and self-healing properties [23]. There are many polymers; for instance, pectin is naturally abundant, cheap, water-soluble, and able to yield conductive hydrogels [24]. As a precursor/monomer and part of double network hydrogels, acrylamide (AAm), diallyldimethylammonium chloride (DADMAC), and (3-Acrylamidopropyl) trimethylammonium chloride (APTAC) are found to be eligible for the formation of CH [25,26,27]. There is a need for such a conductive hydrogel that will be affordable to use in flexible and portable devices and be able to revive its properties after breakdown or fracture through its self-healing nature. A possible future direction for research on CHs is to explore the use of other natural polymers, such as alginate, gelatin, and cellulose, as the first or second network components and to optimize the ratio and distribution of the conductive additives in the hydrogel matrix. The effects of external stimuli, such as temperature, pH, light, and electric field, on the properties and performance of the CHs should be further investigated. Furthermore, the biocompatibility, biodegradability, and toxicity of the conductive hydrogels should be evaluated for biomedical applications, such as tissue engineering, drug delivery, and wound healing. However, this review provides a short description of CHs, including their self-healing and stimuli-responsive properties, strong network structure, natural polymer component, hydrogel for 3D printing, and radiation-induced polymerization. This review will be necessary for the readers to acquire a basic knowledge of the mechanism of conductivity, properties, and limitations of single- or multi-network hydrogels where improvement is required. Moreover, the potential of conductive hydrogel is predicted regarding the improvement of properties as well as conductance.

## 2. Conductive Hydrogels

Recently, conductive hydrogel-based devices have gained great attention for their practical applications. A few of them demonstrate multifunctional activities as smart wearable devices. By changing the raw materials and method of preparation, CHs with variable functionalities are manufactured and employed in different types of machinery to fulfill their demands. For example, conductive hydrogels can be prepared by incorporating conductive fillers, such as nanoparticles, nanowires, and nanosheets, into hydrogel networks or by using conductive polymers as the main components or additives [37]. Usually, single-network hydrogels are mechanically weak and less efficient. To overcome the deficiencies of a single network, a second or third network is fabricated on the first network [38]. In Table 2, recent conductive hydrogels prepared from different raw materials and their respective properties are presented. In this part of the article, some of the latest category CHs are presented along with their prospects, limitations, and scope of development.

### 2.1. Self-Healing Conductive Hydrogels

**Figure 1:**
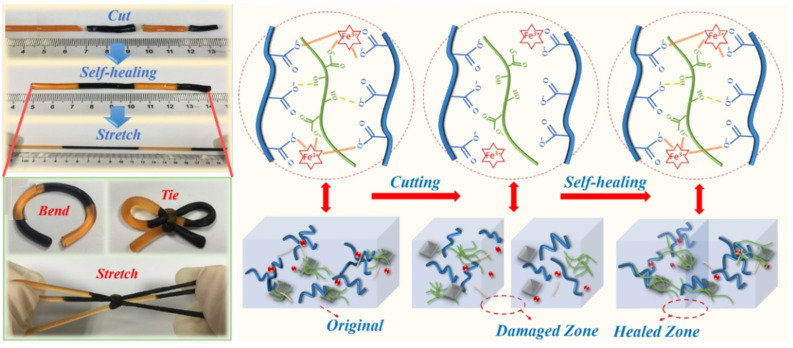
Self-healing mechanism of hydrogels.

The self-healing hydrogels can intrinsically and automatically heal the damages that occurred accidentally or willingly, which is surprising when applied as a conductor in flexible devices. The self-healing ability can enhance the biocompatibility, durability, and adaptability of the hydrogels in the biological environment [64]. Moreover, the self-healing hydrogels can be used as bio-inks for 3D printing of complex structures and organs [65]. The self-healing stretchable CHs are getting much attention day by day due to their efficiency in wearable strain sensors needed for monitoring human health and robotics. There are two types of self-healing hydrogels: (i) covalent bonds, which include imine bonds, disulfide bonds, hydrazine bonds, borate bonds, etc.; and (ii) non-covalent bonds, which include metal coordination bonds, π–π stacking host–guest interactions, and hydrogen bonds [66]. For its self-healing characteristics, the hydrogel should possess both physical (hydrogel bonding) and chemical (ionic interaction) cross-linking. In this case, graphene-incorporated nanocomposites play an important role. The reversible host–guest interaction (hydrogen bonding and ionic interaction) between metal ions (Fe^3+^ or Ca^2+^) and ligands (-COOH or other anions) is the typical self-healing mechanism of the hydrogels shown in Figure 1. Chunxiao Zheng et al. formulated the self-healing hydrogels by dispersing TEMPO-oxidized cellulose nanofibers (TOCNFs)-graphene (GN) nanocomposite into the polyacrylic acid (PAA) [67] and found excellent results listed in Table 3.

**Figure 2:**
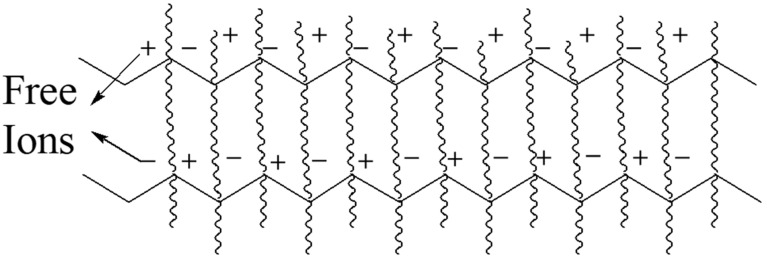
Electricity conduction by conductive hydrogel network.

The transmission of electrons or ions generates conductivity throughout the conductor. The CHs contain polyelectrolytes in their structure and water in a swollen state where the free charges are moving to conduct electricity upon the exertion of external electricity. The common electron-conductive inorganic materials are iron (Fe), gold (Au), silver (Ag), and copper (Cu), and the organic materials are carbon nanoparticles (CNPs), polyaniline (PANI), polypyrrole (PPy), poly (3,4-ethylene dioxithiophene): polystyrene sulfonate (PEDOT: PSS), and polythiophene (PTh) [68]. The inclusion of those materials introduces the conductance property of the hydrogel. Recently, chemically modified graphene has been incorporated into the preparation of hydrogel to increase mechanical strength and conductivity [67]. The organic–inorganic framework part of the hydrogels contains metal ions and counter-negative ions, which allow free electron motion during conduction. Moreover, the delocalized π-electrons on the polymer chain can participate in the electron flow through the hydrogel networks (Figure 2) [68].

### 2.2. Double/Triple Network Conductive Hydrogels

**Figure 3:**
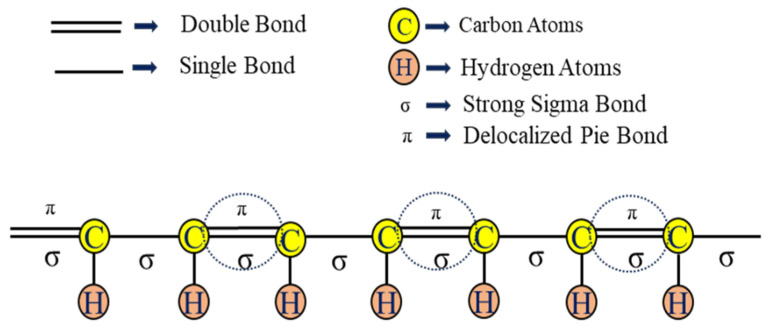
Simplified schematic of a conjugated backbone: a chain containing alternating single and double bonds [76].

**Figure 4:**
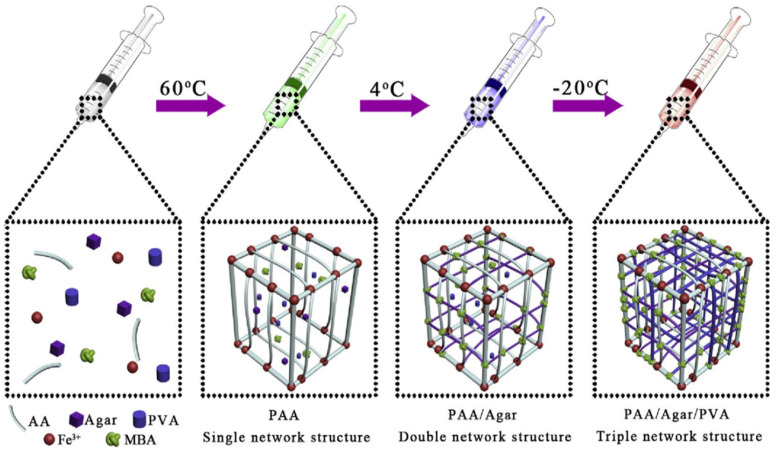
Preparation of Triple network hydrogel [73].

**Figure 5:**
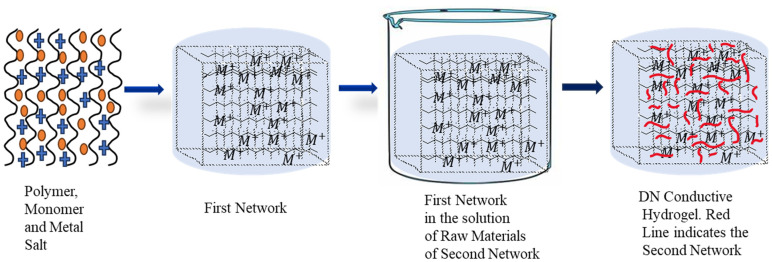
Classical method of DN hydrogel preparation.

Double-network hydrogels are a kind of hydrogel with an interpenetrated polymeric network structure and extraordinary toughness and strength. In the DN gel, two types of networks—(i) the rigid and brittle polyelectrolyte part and (ii) the soft and highly stretchable part—interpenetrate with each other [22]. When the DN gels possess conductive organic polymers or their derivatives in their structure, they are referred to as conductive DN gels. Conductive DN gels are now used in smart textiles, protecting clothing, touch screens, and smart electronics [19,69]. The presence of conjugated double bonds (π–electrons) in the organic polymer molecule can participate in an oxidation-reduction reaction that resembles p-doping and n-doping (Figure 3). As a result, the movement of electrons takes place through the delocalized double bond to form the polymer conductor. To make the hydrogel’s mechanical strength superior, a 3rd polymeric network is brought into the hydrogel along with the double network, which is referred to as a triple network (TN) hydrogel. In other words, triple network (TN) hydrogels are a kind of hydrogel that has three interpenetrating polymeric networks, which can enhance the mechanical strength and toughness of the hydrogel. The third network can be either physical or chemical and can provide additional functions such as self-healing, shape memory, and stimulus responsiveness. TN hydrogels are promising materials for various applications, such as biomaterials, flexible electronics, and smart devices [70]. For example, Wang et al. prepared a TN hydrogel by incorporating poly (vinyl alcohol) (PVA) into the networks of polyacrylamide/polyacrylic acid (PAM/PAA) through a one-pot, two-step method. The PVA network served as a physical cross-linker that improved the stability and elasticity of the hydrogel. The TN hydrogel also showed a fast recovery of mechanical properties after deformation due to the reversible coordination bonds between PAA and Ca^2+^ [71]. In 2014, Okay et al. prepared TN hydrogel based on polyacrylamide and poly (N, N- dimethylacrylamide) with higher mechanical strength [72]. In polyacrylic acid-agar-polyvinyl alcohol (PAA-Agar-PVA) TN hydrogel, agar and PVA act as the second and third networks, respectively, with the first network being PAA-Fe^3+^ (Figure 4), where the inclusion of the Fe^3+^ ion makes it self-healing [73]. The inclusion of conductive monomers or organic materials may enable the hydrogel to be conductive. There are several methods of DN conductive hydrogel preparation; among them, the classical method (Figure 5) can be more acceptable for gamma radiation-induced hydrogels [74]. In this method, a mixed solution of polymers, monomers, and metal salt is polymerized into the first network CH, followed by soaking and swelling in the solution of the second network, which is then subjected to irradiation from a gamma source, resulting in the formation of DN gel [75].

### 2.3. Natural Polymer Based Conductive Hydrogels

**Figure 6:**
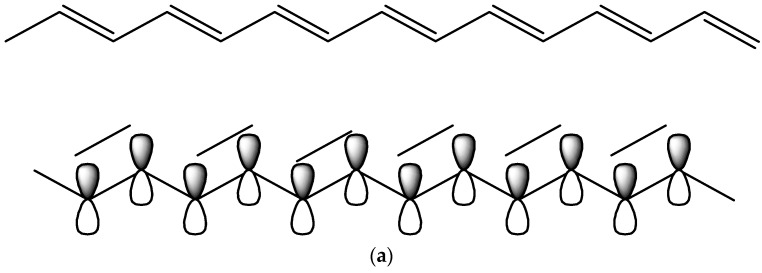

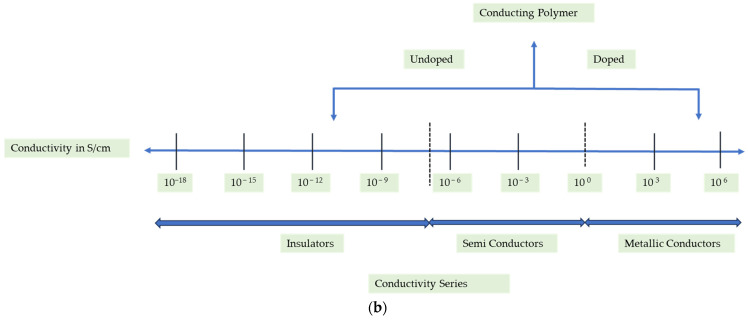
(**a**) Conjugated system of conductive organic compound and (**b**) conductance ranges [86].

Impelling natural polymers into the conductive hydrogel preparation could be a cost-effective step for the device industry. For preparation and application, natural polymers have some advantages over other materials. Due to their biocompatibility, biodegradability, and hydrophilic properties, natural polymer-based CHs are immense candidates for neuron, muscle, and skin-tissue engineering. Considering the best applicability, the most usable natural polymers for CHs are hyaluronic acid, alginate, chitosan, cellulose, collagen, and gelatin. Among them, hyaluronic acid (HA) undergoes direct physical cross-linking through the freeze–thaw method, where there is no need to use an organic solvent and a cross-linking agent [77]. Anionic polysaccharide alginate is capable of forming hydrogels through ionic interaction between cationic divalent or trivalent metal ions (Ca^2+^, Zn^2+^, Ba^2+^, Al^3+^) and carboxylate parts, which facilitates its hydrogel to exhibit electroconductivity [78,79]. Chitosan linear polysaccharide is the second most available (after cellulose) natural biopolymer that forms a non-toxic, pH-responsive hydrogel that can be used for electrodeposition on electrodes [80]. Protein-type polymers, such as collagen and gelatin hydrogels, are thermally unstable as well as weakly chemically cross-linked, discouraging their use as efficient conductive gels [81]. The lack of natural polymer-based CHs is due to their mechanical strength, excess degradation, and low conductance. Researchers are making attempts to mitigate the limitations by modifying the methods of preparation and fabrication of polymers. The electroactive materials are blended, doped, or chemically fabricated on the natural polymers to make them super-active functional materials as conductive gels [82]. Xiangto Liang et al. reported cellulose-based CH with 7.83 × 10^−3^ S/cm conductance along with cellulose-concentration-dependent equilibrium swelling. The polypyrrole (PPy) conductive component was fabricated with microcrystalline cellulose dissolved in an ionic liquid, where the conductivity depends on the doping intensity of TsONa [83]. Xingyue Sun et al. prepared a mechanically improved double network conductive hydrogel by soaking gellan gum/gelatin composite in a solution of NaSO_4_ and (NH_4_)_2_SO_4_, where the chain entanglement improved in the presence of SO_4_^2−^ and Na^+^ ions [84]. Among the monomers and grafting materials, aniline is mostly studied due to the following advantages: being cheap, easily available, stable, inexpensive, compatibility with natural polymers, and better electrical conductivity [85]. When metals allow the delocalization of their outermost electrons, electricity begins to conduct. Similarly, the alternative single and double bonds lying on the polymer backbone enable the materials to be good conductors. Fabrication or doping of conducting monomers extends their conductance (Figure 6).

### 2.4. Stimuli-Responsive Conductive Hydrogels

Hydrogels containing conductive polymers (polyaniline, polypyrrole, polythiophene, etc.) or metals (Fe^3+^, Al^3+^, etc.) that can also show responsivity to pH, temperature, magnetic and electric fields, electromagnetic radiation, and ionic salts are classified as stimuli-responsive hydrogels. Due to both their hydrophilic and conductive properties, stimuli-responsive CHs are widely used in sensors, biomedical fields, and drug release [87]. Owing to its exceptional temperature sensitivity, N-isopropylacrylamide (NIPAM) shows a better temperature stimulus among the CHs. To prepare its CHs, PANI and PPY can be incorporated with NIPAM through a phytic acid cross-linking agent that has six cross-linking points [88]. Along with the conductive polymers, if the hydrogel contains carboxylic groups (–COOH), then it becomes pH-responsive [89,90]. Usually, the graphene sheet or graphene oxides (GO) (as conductive material) are polymerized with acrylic acid to prepare the CHs. Other materials, such as ethylene glycol, dimethylacrylate, hydroxymethyl acrylate, etc., may also be adjusted with those raw materials to improve their conductivity as well as their mechanical properties [91]. The CHs also exhibit responsivity to electromagnetic radiation like near-infrared light (NIR). A few hydrogels are available that show this responsivity, but their mechanical strength and tensile/compressive performance are not satisfactory [92]. However, NIPAM-based polymers, for example, NIPAM-Co-PAM/GO hydrogels coated with arginine–glycine aspartic acid, possess better mechanical strength and responsivity toward NIR light [93]. The hydrogels prepared from electrolytic monomers poly(2-acrylamido-2-methylpropanesulfonic acid (AMPS)), reduced GO, PF-127-diacrylate, and acrylamide can be responded to by an electric field, making them eligible for sensors and actuators that make them capable of controlled drug delivery to the human body [94]. Pressure and tensile force are common stimuli for many hydrogels with adhesive, conductive, stretchable, and toughness properties [95]. To prepare pressure-responsive CHs, the following monomers and polymers have already been used by researchers: acrylate adenine, acryloyl chloride, sodium dodecyl sulfate, hexadecyl methacrylate, polyaniline, pyrimidinone, 2-ureido-4 [1H] pyrimidinone, and poly(4-styrenesulfonate). The self-healing property facilitates the hydrogels being pressure-responsive perfectly. In that case, the hydrogels need to be processed by involving metal salts or electrolytes (LiCl, Borax, Fe^3+^, Ca^2+^) [96]. By including all or most of the stimulus parameters, the multi-stimuli-responsive hydrogels can be designed for diverse applications. Already, a few attempts have been made to prepare and implement these hydrogels for human motion sensors. Zexing Deng et al. reported a multi-stimuli-responsive hydrogel prepared by polymerizing NIPAM with nanoclay and CNT PF127 that simultaneously reflects the responsivity to temperature, pressure, and NIR light, along with the limitations of slow response and weak self-healing nature [97]. Another pH- and electric-field-responsive CH was prepared by the reaction between chitosan-grafted polyaniline and NaIO_4_-oxidized dextrin and successfully applied in drug delivery. However, the tensile strength and compressive mechanical properties were not studied well [98]. Hailong Huang et al. developed Zwitter ionic multi-responsive conductive hydrogel as an E-skin containing a double network polymeric structure of NaCl/sodium alginate/polyacrylate-acrylamide. The hydrogels exhibit excellent sensitivity to strain stress, human motion, and physiological signals, as well as lacking self-healing properties [99]. A host α cyclodextrin-N-isopropylacrylamide and guest PANI CH were prepared without incorporating any metallic components, which limits its properties and multi-stimuli [100]. To overcome the limitations and disadvantages of the existing hydrogels, suitable reagents and methodologies should be searched for and recommended.

### 2.5. Gamma Radiation-Induced Conductive Hydrogels

**Figure 7:**
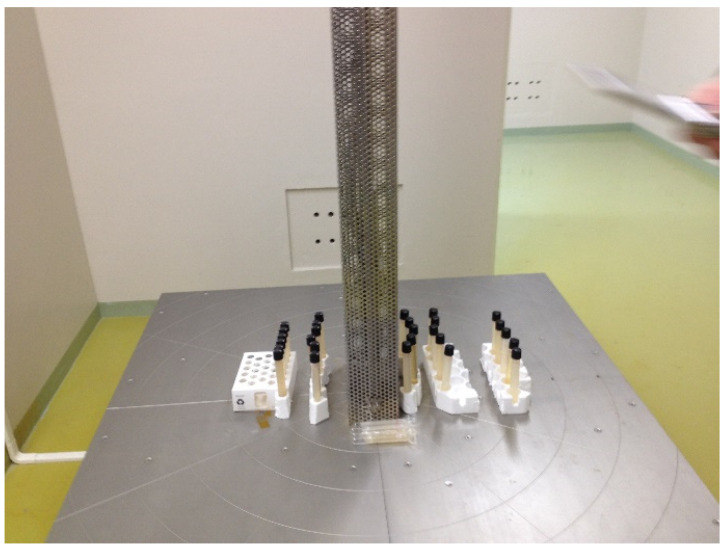
Hydrogel preparation by applying gamma radiation.

There are several methods of preparation of CH, including chemical, electrochemical, and radiation polymerization methods [23,76]. The latter one is timesaving, easy to control, eligible for room temperature and pressure, gives a high yield with purity, and is cheap in industrial production without a chemical initiator or cross-linking agent. Moreover, this technique can induce cross-linking of polymer chains and the incorporation of conductive fillers, such as carbon nanotubes, graphene, or nanosilver [101]. A gamma ray is a high-energy electromagnetic radiation (<0.25 Å wavelength, >12 EHz (1 EHz = 1018 Hz), and >50 keV energy) that can break the bond of monomers easily to yield free radicals and initiate polymerization. The gamma source is a point source where the radiation dose depends on the irradiation time and the distance between the sample vial and the gamma source [102,103]. The author worked on this field, which is presented in Figure 7.

_27_Co^59^ + n → _27_Co^60^ → _28_Ni^60^ + e^−^ + *v*^−^_e_ + gamma rays

This technique is widely used for the preparation of different types of hydrogels, including conductive ones. The conductive polymers PPy, PANI, PVP, and NIPAM can be fabricated into effective CHs by applying gamma radiation. In light of conductance and strength, the PPy hydrogels have some disadvantages, and the latter provide reliable and efficient hydrogels when incorporated with other monomers/polymers and metal ions [103,104]. The PVP is a biocompatible polymer unable to conduct electricity. When 0.15 M Pyrrole in 0.1 M para toluene sulfonate was mixed with 20% PVP and irradiated with a 25 kGy gamma radiation dose, PPy/PVP CH was produced, which exhibits 13.72 ± 3.77 mS/cm conductivity. However, this hydrogel is limited to weak mechanical strength and brittleness [105]. To use in a flexible device, triple network conductive hydrogels were prepared from polyvinyl alcohol/polyethylene glycol diacrylate/agar/sulfuric acid by applying gamma radiation from the ^60^Co source, where the inclusion of sulfuric acid improves the properties of the hydrogel. The conductivity value of this hydrogel is 17.1 mS/cm, which increases to 71.4 mS/cm upon compression to 50%, demonstrating its applicability in strain sensors and ionic cable [106]. The involvement of metal ions in the hydrogel may improve properties like conductivity and fast self-healing. However, there are also some challenges and limitations to this method, such as the high cost, safety issues, and possible degradation of the hydrogels [107,108]. Therefore, further research and optimization are needed to improve the performance and feasibility of gamma radiation-induced self-healing CHs.

### 2.6. Three-Dimensional Printable Conductive Hydrogels

**Figure 8:**
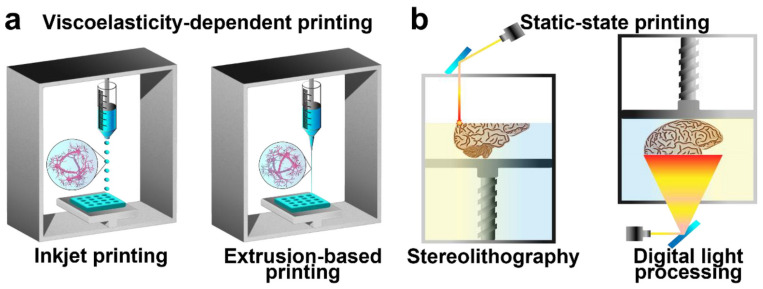
Three-dimensional printing conductive hydrogels (**a**) inkjet or extrusion: conductive hydrogel ink, (**b**) conductive hydrgel ink for printing through photo-polymerization (reused with permission).

The application of additive substances in the production of hydrogels involves the progressive layering of hydrogel material to create complex three-dimensional frameworks through a 3D printer, commonly known as the 3D printable hydrogel technique, and the produced material is called 3D printable hydrogel. This systematic, layer-by-layer technique enables the precise fabrication of complex structures. The first 3D printing technology is stereolithography, which was invented in 1981 [109] and was used commercially around the late 1980s [110]. In Figure 8**,** the types of printing of conductive hydrogels are shown, where hydrogel ink is used for printing as well as printing by photopolymerization reaction [111].

After that, a wide number of studies were carried out to improve and simplify the process to include this technique for the formation of CHs. The objective can be achieved by utilizing different technologies in the field of 3D printing, such as stereolithography (SLA) [112,113,114], extrusion-based printing [115,116,117], and inkjet-based printing [118]. This allows for customization based on specific requirements in tissue engineering [119,120,121], regenerative medicine [122,123], wound healing [124,125], dental application [126], soft robotics [127], and other research activities. In the tissue engineering field, the technique of 3D printing hydrogels is frequently denoted as bioprinting. The fundamental goal of bioprinting is to generate structures that emulate biological tissues and organs. It also facilitates the creation of intricate and tailored constructs for the field of regenerative medicine. The decision regarding the hydrogel material holds immense significance and relies heavily on the intended application. Alginate, agarose, gelatine, and several synthetic polymers are commonly utilized to produce 3D printable CH. However, it is not suitable for hydrogel use in light nervous tissue, which shows less affinity to water, less additivity, and high mechanical stiffness due to conductive materials [128,129,130]. The conductivity of 3D printable hydrogels can be obtained by using the pure conductive polymer PEDOT:PSS with the addition of acid [131], polyethylene glycol diacrylate (PEGDA) [132], ionic liquid [133], and secondary dopants [134,135]. Also, this property can be achieved by applying metals to the hydrogel polymer matrix. A frequently used metal is gold, as it poses more biocompatibility than other metals [136,137]. Rather than gold, other metals, namely silver [138], iron [138], and eutectic gallium indium (EGaIn) [139], are being used. Carbon nanotubes (CNT) and graphene can also be used to serve the purpose of conductivity, though there are problems considering immiscibility and clustering, which have been stabilized by polydopamine coating [140,141], amphiphilic cellulose nanocrystals [142], and silk sericin [143].

A dual or triple network is essential to enhance the hydrogel matrix’s structural stability and high water content. The additional cross-linked network prevents it from dissolving in water, making it suitable for energy applications like zinc/iron micro-batteries [144] and supercapacitors. Cheng et al. successfully synthesized double network cross-linked PEDOT: PSS hydrogel by a 3D printing technique, showing high conductivity (≈3000 Sm^−1^), tough mechanical stretchability (≈55%), high resilience, and low Young’s moduli (≈2.8 Mpa), which can be used in flexible supercapacitors [145]. Zhou et al. fabricated a next-generation flexible supercapacitor that relies on a MXene and PAM (CNF/PAM) dual network hydrogel through 3D printing that shows high areal capacitance (435 mF cm^−2^ at 1 mA cm^−2^), favorable rate capacity (270 mF cm^−2^), energy density 21.7 µW h cm^−2^ at 0.3 mW cm^−2^, and long term reusability of 88.6% over 5000 cycles [146]. Xuran et al. developed a triple network direct-ink-write (DIW) 3D printed PANI/PVA/CNC (PPC) hydrogel that shows a suitable conductivity of 3.35 S m^−1^ for their uniform dispersion of PANI within the hydrogel matrix [147].

In the area of 3D printing, the fabrication of hydrogels poses various challenges that require thorough investigation and resolution. These challenges involve the optimization of printing parameters, the attainment of suitable mechanical properties, the stability of scaffolds, and the preservation of cell viability, especially in the context of bio-printing [148]. To overcome these hurdles, researchers are actively engaged in addressing these challenges, aiming to drive progress and enhance the capabilities of 3D printing in hydrogel applications. The utilization of nanoparticles in hydrogels has opened up new possibilities for development in this area that possess unique properties and serve specific purposes like enhancing mechanical, electrical, and biological characteristics. Nowadays, organic and inorganic nanoparticles including polymers, carbon-based materials, ceramics, metals, and metal oxides are being used [149]. After preparing the precursor for hydrogels, conductive additives need to be included. Matrix design is then implemented through computer-aided design (CAD) software, version 24.2). The conductive layer might be coated after the formation of hydrogel in some cases.

## 3. Current Challenges and Future Prospects

The lack of some characteristics in the CHs demands modification and fabrication to make them acceptable in the field of application. In recent years, research on conductive hydrogels has blossomed as the area of applications is vast. It may be widely used in the field of intelligent electronic devices in the near future. Remarkable progress has been made in this field of material synthesis and optimization, but it still lags behind in practical applications. Most of the synthesized CHs can detect human motions, but in a few cases, it is important to monitor sweat and temperature as they reveal important information about health conditions. Xu et al. [150] developed a tannic acid-Ag-CNT-PANI composite conductive hydrogel that can detect pH and tyrosine in human sweat. However, other properties, like conductivity, did not show a notable amount. So, without sacrificing other important properties, it is essential to develop CHs with different and price-effective materials for monitoring human sweat as well as temperature. When hydrogels are submerged in water or exposed to high humidity, the unequal osmotic pressure leads to water uptake, resulting in a decrease in their mechanical properties. This limitation hinders their potential applications and shortens their lifespan. To address this issue, future research should focus on optimizing hydrogel materials to prevent water volatilization, especially in hot or dry conditions. One effective approach is to select high-performance water-retaining agents to enhance the water retention capacity of hydrogels. Additionally, the absorption and volatilization of water can also lead to changes in the shape and size of hydrogels, which poses a significant challenge for the development of micro-hydrogel devices. In recent times, different research has been carried out for the sustainability of CHs in anti-freezing and hot temperatures, but it needs to be extended as the research has not evaluated all the possible outcomes [45,62]. The anti-biofouling and self-cleaning properties of CHs need to be further investigated, as they depend on the other properties too. As the main property of this hydrogel is conductivity, it should be the main focus to enhance this without affecting other properties like mechanical, thermal stability, etc. Electron conduction and ionic conduction are the main two mechanisms for the conductivity of a hydrogel. Again, electronic conductivity depends on two methods: (i) CHs formed directly from conductive polymers; and (ii) combining micro- and nanomaterials into the polymer matrix, where excessive addition leads to lower mechanical properties [151]. On the other hand, for ionic conduction, ions need free movement inside the matrix. However, water retention affects ion movement, especially in hot or cold temperatures.

A few of the previously studied CHs, with their respective conductance, are listed in Table 4.

To use in flexible devices and biomedical fields, the DN/TN CHs will be the pioneer candidates shortly. In the past few years, researchers have tried to develop multi-stimuli-responsive CHs. Still, there are significant updates required, such as fast self-healing, sufficient conductivity and thermal stability, flexibility, etc. Two of the challenging factors are the combination of raw materials and preparation methods. If the application destination of CHs is in the human body, the product and execution results should be as precise and accurate as possible. One should select or prepare such a raw material that can form self-healing DN/TN CH. Therefore, proper selection of raw materials and preparation methods may provide a better edict to represent all or most of the required demands of smart CHs. It is more acceptable to make the CHs a double or triple network where the first part or network (made of a conductive organic compound) is self-healing as well as conductive, and the second part or network is highly conductive. Due to its unique properties, PANI is a promising conductive polymer for use in diverse fields of application, including wastewater treatment, dye adsorption, and CHs synthesis [85,159]. Along with hyaluronic acid, natural polymer pectin plays a vital role in forming CHs and flexible electronics [160,161]. Based on the overall discussion on raw materials, the author predicted the combination of organic conductive materials in the first and second networks for multi-stimuli CHs, as shown in Table 5. From the experimental data, the best one can be optimized for use on various devices.

## 4. Conclusions

Conductive hydrogels are pioneering materials used in different devices and instruments due to their excellent flexibility, versatility, and biocompatibility. Even recently, hydrogels have been used as printable conductors and metal-free batteries. In this review, we aimed to provide a comprehensive overview of the recent advances and prospects of CHs. We first introduced the basic concepts of CHs as well as the types of conductive materials and crosslinking methods used to prepare CHs. Different conductive organic compounds and polymers are fabricated together and produce desirable hydrogels. The stimuli-responsivity properties are tuned to make the hydrogel suitable for various devices like sensors, actuators, flexible energy storage, solar cells, and soft robotics. To extend the mechanical strength as well as conductivity, the single-network hydrogels are turned into double- or triple-networks. The self-healing property has brought tremendous changes to hydrogels and broadened their field of application.

Here, the self-healing and electrical conduction mechanisms, natural polymer-based CHs, and 3D printable CHs were reviewed and analyzed with relevant figures. We listed some of the recent CHs with their properties, conductance, applied fields, and predictions of probable combinations of raw materials suitable for the preparation of CHs. Three-dimensional printing with conductive hydrogels is the revolutionary application being used in the present printing section. Finally, we conclude with some remarks on the current limitations and future directions of CH research. We hope that this review can inspire more innovative ideas and interdisciplinary collaborations in the field of CHs.

## Figures and Tables

**Table 1:** A few conductive organic compounds/polymers and their properties.

S.N	Conductive Organic Compounds /Polymers	Structure		Properties	Reference
1	TCNQ	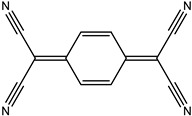		(i) Good electron acceptor and donor (ii) Possesses multi-redox properties (iii) Can form charge transfer metal-complex	[28]
2	TTE	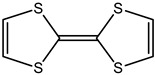		(i) Good pie electron donor (ii) Can reversibly transform into TTF^+^ and TTF^2+^	[29]
3	BEDT-TTF	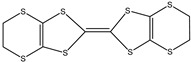		(i) Can form a superconductor (ii) Organic donor molecule	[30]
4	PANI	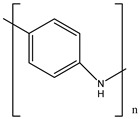		(i) Can undergo doping with various acids (ii) High conductivity (iii) Low toxicity	[31]
5	PPy	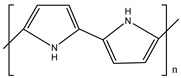		(i) Positively charged heterocyclic polymer (ii) Electroactive in aqueous solution (iii) Possesses redox properties	[32]
6	PEDOT	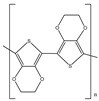	PEDOT: PSS	(i) These two form semiconductors through doping (ii) Strong electrical conductivity (iii) Excellent oxidation resistance	[33]
7	PSS	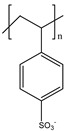			
8	PTh	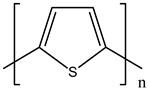		(i) Show conductivity through pie conjugation (ii) Potential for optical and electronic devices	[34]
9	PPV	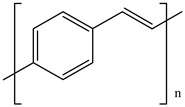		(i) Rigid-rod-like polymer (ii) Can form a thin crystalline film (iii) Possesses optical band gap	[35]
10	PC	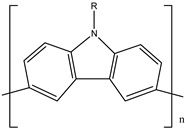		(i) Optoelectrically and morphologically stable (ii) Good for nanodevices and rechargeable batteries	[36]

**Table 2:** A few recent conductive hydrogels and their properties.

S.N.	Conductive Hydrogels	Notable Properties	Field of Application	Reference
1	Carboxymethyl cellulose/poly(acrylic acid/Fe^3+^/LiCl	(i) Stretching—recovering range up to 400% (ii) High conductivity of 5.89 S m^−1^ (iii) High sensitivity (gauge factor, GF = 6.19 at 200–400% of strain) (iv) Single electrode capacitance of 122.36 F g^−1^ (v) Good anti-freezing capacity	Flexible electronics	[39]
2	PVA/PA/PDA	(i) Elongation breakup of 550% with tensile strength of 234 kPa (ii) Self-adhesivity up to 14.9 kPa (iii) Strain coefficient GF = 3.67 at 150–200% of strain (iv) Conductivity of 2.21 S m^−1^.	Wearable bioelectronic sensors	[40]
3	Acrylamide/Lauryl methacrylate/L-glutamic acid	(i) 1500% manual stretchability (ii) High toughness of 740 kJ m^−3^ with Young’s modulus of 1.65 kPa (iii) High GF of 9.42 and conductivity of 0.28 S m^−1^	Wearable strain sensors	[41]
4	ε-PL-SH/PPy (CHLY Collagen based hydrogel)	(i) Adhesion strength of 2.36 kPa (ii) Compressive strength of 45 kPa and conductivity of 3.167 S m^−1^ (iii) Hemolysis ratio (HR) and blood clotting index (BCI) is 0.6% and 21.7%, respectively (iv) High tissue adhesion ability, low cytotoxicity	(i) Wound healing (ii) Tissue engineering	[42]
5	PVA/CMC/LiCl	(i) Moderate voltage, current, and power density of 584 V, 25 µF, 25 W/m^2^, respectively (ii) Conductivity of 0.4 S m^−1^	Touch sensor	[43]
6	PVA/PEDOT:PSS/NaCl	(i) Resistance charge up to 20% and maximum strain of 223% (ii) Thermal stability up to 353 °C (iii) High tensile strength of 0.23 MPa (iv) Elongation break up at 233% strain, GF = 0.41 at 100% strain (v) Fast response time of 0.88 s	Wearable sensors	[44]
7	PVA/TEF/SF	(i) Notable elongation of 1107.3% (ii) High healing rate at 91.11% (iii) Anti-freezing capacity up to −41 °C (iv) Compressive strength of 153.35 kPa, tensile strength of 126 kPa, GF = 6.32 at 150% of strain	Wearable sensors	[45]
8	NIPAM/co-MBAA/AM with ionic LiCl and glycerol	(i) High thermal sensitivity 5.51%/ °C (ii) High resolution of 0.2 °C and high transparency of 92% (iii) High stretchability of 1972% with high gauge ratio of 17.3	(i) Flexible sensor (ii) Soft robot	[46]
9	PVA/EG(ethylene glycol) with metal ion MgCl_2_	(i) Tensile strength of 1.1 MPa with 442.3% of elongation break up (ii) Anti-freezing stability up to −20 °C (iii) Competitive GF value of 0.725 of relevant strain (iv) Conductivity is 0.172 S m^−1^	Flexible strain sensor	[47]
10	Agar/Borax/MXene	(i) High conductivity of 8.14 S m^−1^ with 129 kPa strength (ii) Elongation break up at 105.1% of strain with GF up to 1.52	Flexible strain sensor	[48]
11	AAm/co-Butyl acrylate/Gaur Gum	(i) High toughness of 76 kJm^−1^ (ii) Conductivity of 0.2 S m^−1^ (iii) GF is 8.2 at 400% of strain	(i) Motion monitoring (ii) Wearable electronic devices	[49]
12	P(AM-APBA)XLG/CNTs	(i) Tensile strength of 252–323 kPa, fracture strain range to 880–1200%, Young’s modulus of 48–50 kPa (ii) Remarkable sensing performance (as GF up to 9.43 with relevant strain)	(i) Flexible wearable devices (ii) Healthcare devices	[50]
13	PAM/SA/CNTs with silica	(i) Good tensile strength ~291.6 kPa and tensile strain ~589.7% (ii) Improved GF = 2.6–3.1 with strain range of 100–200% (iii) Withstand ability to temperature of −20 °C	(i) Wearable electronic devices	[51]
14	Bovine serum albumin-MA-PPy/P(AM-co-AA)/Fe^3+^	(i) High tensile strength 5.36 MPa and toughness 17.66 MJ/m^3^ (ii) Elastic modulus 1.62 MPa (iii) Fast self-recovery 99.89% (iv) Conductivity of 1.14 S m^−1^ (v) High strain sensitivity as GF is 4.98 (vi) Good biocompatibility	(i) Soft electrode in electrocardiogram device (ii) Strain sensors (iii) Biosensors (iv) Bioelectronics	[52]
15	PVA/PAAm/XG (xanthum gum)/Zn^2+^	(i) Capacity of the supercapacitor retains at 88.24% after 10,000 cycles (ii) High current density 10 A g^−1^ (iii) Tensile strength 1.14 MPa (iv) Fracture strain of 603% with toughness of 2.9 MJ m^−3^ (v) Conductivity of 3.098 S m^−1^ (vi) Anti-freezing stability up to −60 °C, strain sensitivity high (GF = 4.40)	(i) Supercapacitors (ii) Flexible electronic devices (iii) Soft sensors	[53]
16	PEDOT:PSS/CNTs	(i) High electrical conductivity of ~2000 S m^−1^ (ii) Water content capacity 96% (iii) Biocompatibility	(i) Wearable devices (ii) Soft robotics (iii) Bioelectronics	[54]
17	PAAm/PVA/PDA-Fe_3_O_4_-MXene	(i) Tensile strength 156 kPa (ii) Conductivity 0.110 S m^−1^ (iii) Wide working range of strain 3–300% (iv) Rapid response time of 290 ms (v) High sensitivity as GF is 1.16 at 100–300% strain (vi) Toughness of 342.8 KJ m^−3^	(i) Strain sensors (ii) Wearable devices	[55]
18	KMGHCa (K-MXene/GG/HEAA/CaCl_2_)	(i) Tensile stress of 1463% and tensile strain of 1008 kPa (ii) Electrical conductivity of 2.07 S m^−1^ (iii) Anti-freezing ability (iv) High sensing range 0–400% with high sensitivity at a GF of 4.4 (v) Photothermal conversion efficiency of 93.6% (vi) Thermoelectric sensitivity of −0.41%/°C	(i) Self-powered triboelectric nanogenerators (ii) Photothermal detector (iii) Monitoring sensors (iv) Energy harvesting devices	[56]
19	AG/SBMA/PPy with Fe^3+^	(i) Moderate sensitivity with GF 3.096 for 0–10% strain ranges (ii) Resistance to swelling underwater	(i) Wearable electronic devices	[57]
20	P123(Pluronic)/LAD/TMAx	(i) Ionic conductivity 0.18 S m^−1^ (ii) Stretchability up to 1611% (iii) Remarkable sensitivity as if GF is 4.98 at 500% strain and wide strain range 0.1–500%	(i) Flexible sensors (ii) Electronic skin devices (iii) Biomedical devices	[58]
21	PVA/AAc/NaCl	(i) Elongation up to 550% of strain (ii) Compression modulus 9.25 kPa (iii) Gauge factor of 2.29 at 100–300% of strain	(i) Supercapacitor (ii) Energy storage	[59]
22	Chitosan(CS)/tannic acid(TA)/PAA (QCMCS hydrogel)	(i) High nonlinearity of GF 2.05 with a large strain 20–1400%, strength 169 kPa, tensile strength 169.4 kPa, toughness 1069.9 kJ/m^−3^ (ii) Adhesion strength of 16.2 kPa (iii) Conductivity of 3.8 S m^−1^ (iv) Biocompatibility and Antibacterial	(i) Tissue engineering, (ii) Artificial conductive skin (iii) Wearable devices and strain sensor	[60]
23	CS/CSF_1.5_-PAA-Fe^3+^-G hydrogel	(i) Tensile strength 173.9 kPa, elongation break up at 1477% of strain (ii) Excellent durability, and stability up to 1000 cycles (iii) Adhesive strength to SS metal is 25.38 MPa (iv) Fast self-healing nature (v) Gauge factor value 2.75 at strain range 250–600%	(i) Monitoring device for human health (ii) Wound healing	[61]
24	HA/MA-rGO-PANI	(i) Thermal stability at high temperatures (ii) Conductivity of 0.00158 S m^−1^ (iii) Compressive strength of 992.11 kPa and elastic modulus of 23.60 kPa	Drug delivery	[62]
25	SA(sodium alginate)/CaCl_2_/AgNO_3_	High surface resistivity	Textile applications	[63]

**Table 3:** Parameter values of TEMPO-oxidized cellulose nanofibers—graphene CHs.

Parameter		Results
Stretchability		~850%
Viscoelasticity (storage modulus)		of 32 kPa
Mechanical strength	Compression strength	2.54 MPa
	Tensile strength	0.32 MPa
Electrical conductivity		~2.5 S m^−1^
Healing efficiency		96.7% within 12 h

**Table 4:** Previous self-healing conductive hydrogels and their conductance/resistance.

S.N	Hydrogel	Conductance	Application	Reference
1	Self-Healing Conductive Injectable hydrogels	2.25–3.5 × 10^−3^ S cm^−1^	Wound dressing and cutaneous, wound healing	[152]
2	Polysaccharide-templated conductive and self-healing hydrogel	1.52 × 10^−3^ S cm^−1^	Circuit	[153]
3	Dual ionic cross-linked double network hydrogel	1.6–6.2 × 10^−3^ S cm^−1^	Self-repaired circuit	[154]
4	Non-covalently Assembled Electroconductive hydrogel	~8–~16 × 10^−3^ S cm^−1^	Tissue engineering	[155]
5	Hydrogel with Super Metal Adhesion	1.05 × 10^−2^ S cm^−1^	Adhesive	[156]
6	Human Motion Sensing hydrogel	1.3–1.9 × 10^−3^ S cm^−1^	Human motion	[97]
7	Multifunctional Stimuli-Responsive hydrogels	3.5 × 10−2 S cm^−1^	Sensors, human motion sensing	[157]
8	Polypyrrole/Agarose-Based conductive hydrogel	1.91 × 10^−6^ −1.95 × 10^−1^ S cm^−1^	Patterning and self-repaired circuit	[158]

**Table 5:** Predicted combination of raw materials in first and second network for CHs.

	First Network	Second Network
A	Hyaluronic acid + FeCl_3_ + Acrylic acid	PANI
B	Pectin + Hyaluronic acid + NaIO_4_	PANI + NIPAM
C	Pectin + Hyaluronic acid + LiCl	NIPAM + Acrylic acid
D	Hyaluronic acid + Alginate + CaO	Tetracyanoquinodimethane + PANI
E	2-acrylamido-2-methylpropanesulfonic acid + Na_2_SO_4_ + PANI	Graphene + NIPAM
F	Hyaluronic acid + Fe_2_(SO_4_)_3_ + Polythiophene	Graphene + Acrylic acid

## Data Availability

No new data were created or analyzed in this study.

## Abbreviations

3D	3-dimension
AAm	Acrylamide
AMPS	Poly (2-acrylamido-2-methylpropanesulfonic acid
APTAC	(3-Acrylamidopropyl) trimethylammonium chloride
BEDT-TTF	Bis(ethylenedithiolo) tetrathiafulvalene
CAD	Computer-aided design
CHs	Conductive hydrogels
CMC	Carboxymethyl cellulose
CNPs	Carbon nanoparticles
CNT	Carbon nanotube
CS	Chitosan
DADMAC	diallyldimethylammonium chloride
DIW	Direct-ink-write
DN	Double network
EGaIn	Eutectic gallium indium
GN	Graphene
GO	Graphene oxides
HA	Hyaluronic acid
NIPAM	N-isopropylacrylamide
NIR	Near-infrared light
PAA	Polyacrylic acid
PANI	Polyaniline
PC	Polycarbazole
PEDOT: PSS	Poly(3,4-ethylenedioxythiophene: polystyrene sulfonate)
PEGDA	Polyethylene glycol diacrylate
PPV	Phenylene vinylene
PPy	Polypyrrole
PTh	Polythiophene
PVA	Poly (vinyl alcohol)
TCNQ	7,7,8,8-tetracyanoquinodimethane
TMTSF	Tetramethyl-tetraselenafulvalene
TN	Triple network
TOCNFs	TEMPO-oxidized cellulose nanofibers
TTE	Tetrathiafulvalene
